# Changes in the malaria transmission profile in a low-endemic area of the Brazilian Amazon: a cause for concern

**DOI:** 10.1590/S1678-9946202567070

**Published:** 2025-10-13

**Authors:** Francisco Marques de Oliveira-Neto, Hermano Gomes Albuquerque, Antonio Rafael da Silva, Eloisa da Graça do Rosario Gonçalves, Martha Cecilia Suárez-Mutis

**Affiliations:** 1 Fundação Oswaldo Cruz, Instituto Oswaldo Cruz, Laboratório de Doenças Parasitárias, Rio de Janeiro, Rio de Janeiro, Brazil; 2 Fundação Oswaldo Cruz, Instituto Oswaldo Cruz, Programa de Pós-Graduação em Medicina Tropical, Rio de Janeiro, Rio de Janeiro, Brazil; 3 Universidade Federal do Maranhão, São Luís, Maranhão, Brazil; 4 Universidade Federal do Maranhão, Programa de Pós-Graduação em Saúde e Ambiente, São Luís, Maranhão, Brazil

**Keywords:** Malaria, Maranhao State, Epidemiology, Surveillance, Elimination

## Abstract

Since 2015, there has been a renewed global commitment to malaria elimination. Understanding the dynamics of the disease in regions with declining cases is essential to prevent reintroduction. This study aims to analyze the epidemiological indicators of malaria in Maranhao, a Brazilian state with a significant decline in cases. This is a descriptive, retrospective, and ecological study, with data from the malaria surveillance system, from 2003 to 2022. Demographic, spatial, temporal, and parasitological variables were analyzed. During this period, 83,517 malaria cases were reported, of which 67.8% were autochthonous, and among these, 83.1% occurred in rural areas. The Annual Parasitic Incidence (API) has recently been considered low risk in most municipalities. The analysis of seasonality was important at the beginning of the series, but with the reduction of cases, it lost relevance. The epidemiological profile has shifted, with an increase in imported cases initially from abroad and more recently from other Brazilian states. There was also a significant reduction in the proportion of *P. falciparum*, from 17.7% in 2003 to 6.01% in 2022. Most infected individuals were male, predominantly aged 15 to 45 years, of which increased from 59.1% to 70.5%. Although the overall trend is downward, recent changes in the disease profile are concerning, as they could reverse progress and cause a new rise in cases in a state close to elimination. Surveillance must be strengthened and adapted to prevent the reintroduction and resurgence of endemic transmission.

## INTRODUCTION

Malaria is one of the most relevant parasitic diseases affecting humans, and is currently endemic in 85 countries^1^. In Brazil, over 99% of malaria cases are concentrated in the Amazon region^2^. However, within this region, incidence is extremely heterogeneous across localities, with increases in areas with high mining activity and in some indigenous lands affected by mining^3^. Although malaria is not one of the main diseases in Maranhao State, it has historical relevance in rural and Amazonian areas of the state, especially in contexts of social and environmental vulnerability^4-6^. From 2000 onwards, there was a steady reduction in cases after the implementation of the Plan for Intensification of Malaria Control Actions in the Legal Amazon (PIACM), followed by the National Malaria Control Program (PNCM), in 2003. In the following years, the gradual decrease in cases continued, maintaining a low-transmission scenario despite minor fluctuations^2,5,6^. Close monitoring is necessary in these areas, as various factors could reintroduce the disease^7^.

In 2015, the World Health Organization (WHO) launched the Global Technical Strategy (GTS), aiming to eliminate malaria worldwide^8^. Brazil embarked on this proposal, initially focusing on eliminating *P. falciparum* malaria, and in 2022, it also proposed eliminating *P. vivax* malaria^9,10^. Understanding malaria dynamics in areas with low or interrupted transmission is crucial to prevent its resurgence and to inform appropriate actions to maintain a malaria-free status. In areas where transmission still occurs, identifying the strategies necessary to fully interrupt transmission and avoid reintroduction is imperative^7,9,10^.

Brazil is one of the countries with a consistent malaria database^11^, and using surveillance data is an important tool to understand changes in transmission patterns. Therefore, regularly updating our epidemiology knowledge is essential to develop data-driven plans that strengthen efforts to eliminate malaria in Maranhao State. This study aims to analyze the epidemiology of malaria in Maranhao and to understand transmission dynamics over the last 20 years, from 2003 to 2022.

## MATERIAL AND METHODS

### Ethics

The study was approved by the Ethics Committee of the Oswaldo Cruz Institute (Fiocruz) under CAAE Nº 60198622.2.0000.5248 (opinion Nº 5.520.249 on 2022/07/11).

### Study area

**Figure 1:**
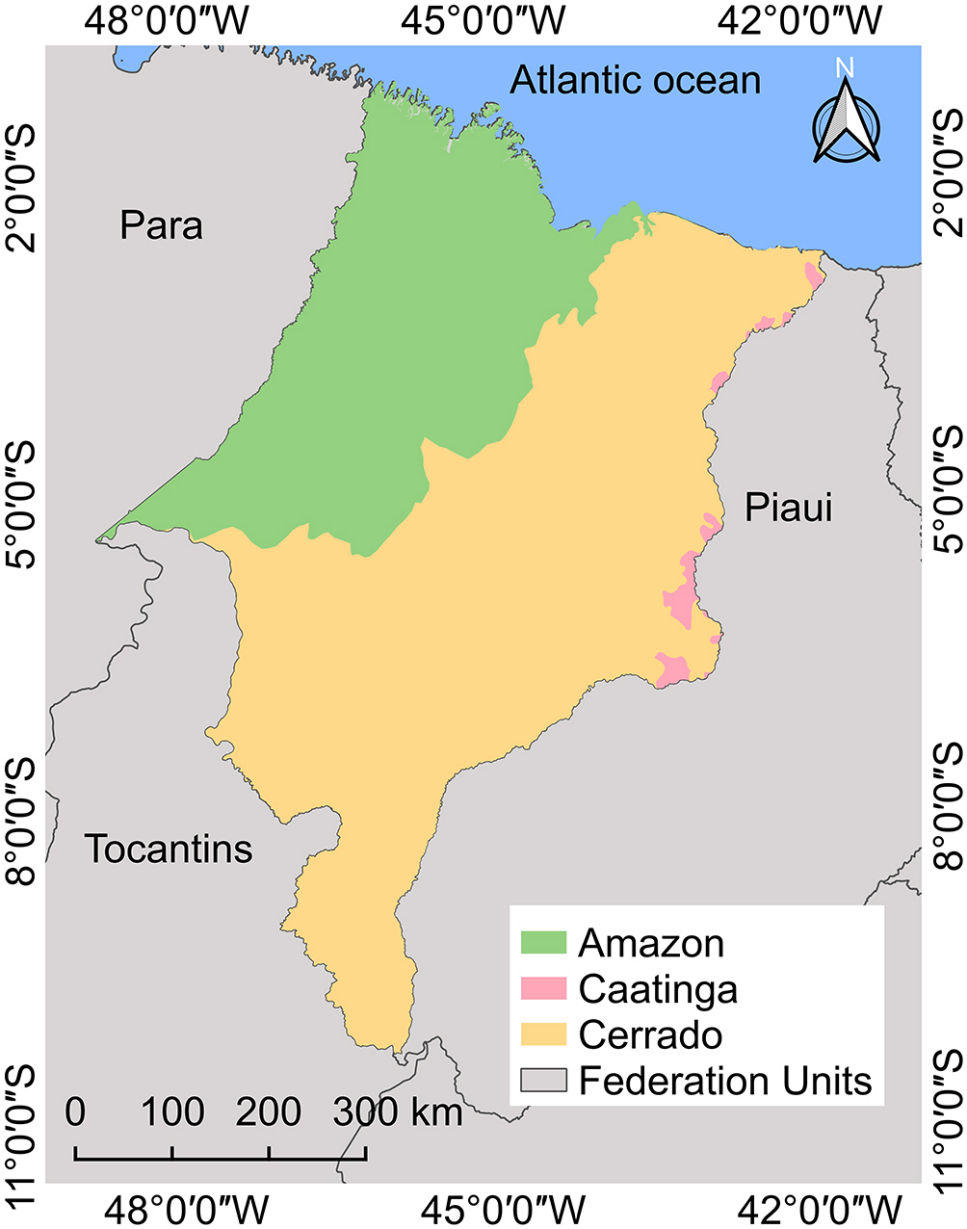
Map of the Maranhao State divided by biomes.

Maranhao State is located in Northeast Brazil and borders three states: Piaui (East), Tocantins (South and Southwest), and Para (West), as well as the Atlantic Ocean (North). It comprises 217 municipalities, covering a territorial area of 329,651,496 km^2^, with a population of 6,775,152 inhabitants and a Human Development Index (HDI) of 0.676, making it one of the poorest states in Brazil^12^. The state encompasses three biomes (Figure 1): Amazon, Cerrado, and Caatinga^13^. The climate is categorized into three main types: equatorial, which is hot and humid under Amazonian influence with high precipitation throughout the year; humid tropical, which covers most of the state and features two well-defined seasons, the rainy season typically extending from January/February to June and the dry season from July to December/February; and dry humid tropical, which also has two defined seasons but experiences a shorter rainy period and a more pronounced dry season due to its proximity to the semi-arid Northeast^14,15^.

### Study design

This is a descriptive, retrospective, and ecological study aimed at analyzing reported cases of malaria in Maranhao State, from 2003 to 2022. In Brazil, all malaria cases are mandatorily reported. Cases occurring in the Amazon region are entered into the Malaria Epidemiological Surveillance Information System (SIVEP-Malaria)^11^. The epidemiological case form includes 54 variables and is filled at the local level for each suspected case. Aggregated information without patient identification is publicly available. For this study, variables corresponding to person, time, place, and parasite species were evaluated. Aggregated data without personal identification of affected individuals (SIVEP-Malaria)^11^ were provided by state health authorities from 2020 to 2023. Only new cases were considered. Thick blood smears used to verify cure after diagnosis (LVC – Lamina de Verificacao de Cura), even if positive, were excluded from the analysis.

### Data analysis methodology

Demographic variables (age and sex), spatial variables (municipality of residence and probable place of infection, urban/rural malaria, imported/autochthonous cases), temporal variables (month and year of infection), and parasitological variables (parasite species) were analyzed descriptively. Categorical variables were assessed using the χ^٢^ test of independence or Fisher’s exact test, as appropriate. Continuous variables were analyzed using Student’s t-test or Mann–Whitney’s test, depending on normality test results. Statistical analyses were performed using the SPSS 23.0 statistical software (SPSS Inc., Chicago, MI, USA). In addition to evaluating the number of cases, the annual parasite incidence (API) was also calculated, defined as the number of new autochthonous cases each year divided by the population of the area, multiplied by 1,000. The endemic curve was obtained by calculating the median number of cases per month for each year, considering the interquartile range (IQR3) as the upper limit and the interquartile range (IQR1) as the lower limit, with epidemic peaks exceeding the upper limit. To analyze the duration of epidemic episodes from 2010 to 2020, the classification described by Braz *et al*.^16^ was used: short-term epidemics (one to four epidemic months per year), medium-term epidemics (five to eight epidemic months per year), and long-term epidemics (nine to 12 epidemic months per year). Cases were classified as autochthonous and imported. Due to the heterogeneity of human and environmental occupation in the Amazon, locations of autochthonous transmission were classified according to the standardization of special areas of the Brazilian Ministry of Health as rural, urban, mining, indigenous, and settlement areas, as these categories guide targeted control actions^9,10,17^. All maps were produced using the QGIS Geographic Information System program. 3.30.1 Hertogenbosch.

## RESULTS

**Figure 2:**
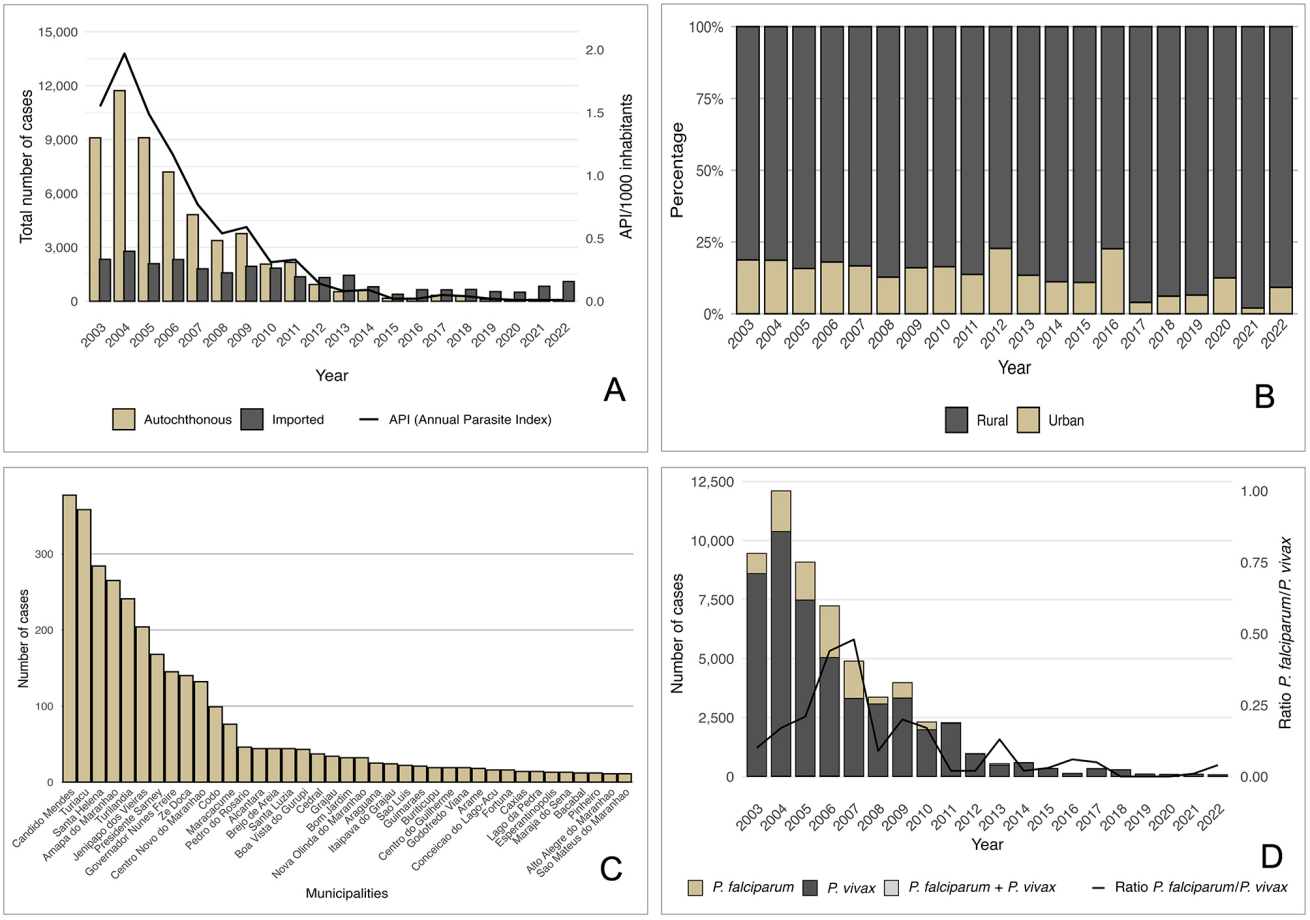
(A) Annual percentage of autochthonous and imported malaria cases recorded in Maranhao State, 2003–2022; (B) Percentage variation between urban and rural cases, 2003–2022; (C) Municipalities that reported more than 10 malaria cases, 2012–2022; (D) Number of cases and ratio of *P. falciparum* to *P. vivax*, 2003–2022.

Results were analyzed separately for autochthonous and imported cases, urban and rural malaria, seasonality, and API, with the latter analyses focusing on autochthonous cases, while sociodemographic and parasitological analyses were based on the total number of confirmed cases. From 2003 to 2022, a total of 83,517 malaria cases were confirmed in Maranhao State, with an average of 4,163 ± 3,460 cases per year (median: 2,109; 95% CI: 1,516–5,679) (Table 1). Two distinct periods were identified: the initial period (2003–2011), characterized by a high and continuous number of cases, and the subsequent period (2012–2022), marked by a sustained decline. This division was based on changes in the epidemiological profile, demonstrated by the consistent reduction in cases from 2012 onwards, providing a more accurate comparison between different scenarios. From 2003 to 2005, the state recorded over 10,000 cases annually. Starting in 2015, the number of cases decreased to less than 1,000 per year. Only 552 cases were reported in 2015, representing a 95.17% decrease from 2003. Although 2003 was the first year of SIVEP-Malaria implementation and may have included underreporting, 2004 registered 9,086 cases, supporting the validity of this comparison. In 2022, 1,172 cases were recorded (Table 1 and Figure 2).

**Table 1:** Distribution of autochthonous and imported malaria cases in a low-transmission region of the Brazilian Amazon (2003–2022)

Year	Autochthonous cases						Imported cases		
	Urban		Rural		Total		Imported		Total
	n	%^*^	n	%^*^	n	%^**^	n	%**	N
2003	1,705	18.73	7,397	81.27	9,102	79.62	2,330	20.38	11,432
2004	2,188	18.66	9,539	81.34	11,727	80.84	2,780	19.16	14,507
2005	1,434	15.75	7,673	84.25	9,107	81.34	2,089	18.66	11,196
2006	1,297	18.01	5,903	81.99	7,200	75.64	2,319	24.36	9,519
2007	803	16.66	4,017	83.34	4,820	72.80	1,801	27.20	6,621
2008	430	12.73	2,948	87.27	3,378	68.16	1,578	31.84	4,956
2009	603	16.02	3,162	83.98	3,765	65.98	1,941	34.02	5,706
2010	338	16.38	1,726	83.62	2,064	52.86	1,841	47.14	3,905
2011	296	13.70	1,865	86.30	2,161	61.39	1,359	38.61	3,520
2012	212	22.77	719	77.23	931	41.41	1,317	58.59	2,248
2013	71	13.42	458	86.58	529	26.85	1,441	73.15	1,970
2014	66	11.15	526	88.85	592	42.41	804	57.59	1,396
2015	18	10.91	147	89.09	165	29.89	387	70.11	552
2016	29	22.66	99	77.34	128	16.69	639	83.31	767
2017	13	3.96	315	96.04	328	34.20	631	65.80	959
2018	17	6.14	260	93.86	277	29.75	654	70.25	931
2019	7	6.54	100	93.46	107	16.74	532	83.26	639
2020	11	12.50	77	87.50	88	14.94	501	85.06	589
2021	2	2.00	98	98.00	100	10.73	832	89.27	932
2022	7	9.21	69	90.79	76	6.48	1,096	93.52	1,172
Total	9,547	16.85	47,098	83.15	56,645	67.82	26,872	32.18	83,517

^*^The denominator is the total number of autochthonous cases; **The denominator is the total number of cases. Autochthonous cases in rural areas include cases in indigenous areas, settlements, camps, riverside communities, and other rural areas.

Of the total accumulated cases, we classified 56,645 (67.82%) as autochthonous and 26,872 (32.18%) as imported (Table 1 and Figure 2). Among imported cases, 39.59% originated from other Brazilian states, while 60.41% came from other countries.

### Autochthonous cases

Autochthonous cases were analyzed separately to describe local malaria transmission patterns in Maranhao State. During this period, most cases occurred in rural areas: 47,098 cases (83.15%), with an annual average of 2,355 cases, while 9,547 cases (16.85%) occurred in urban areas (annual mean: 477 cases). Rural areas recorded 82.95% of cases during the epidemic period (2003–2011), rising to 86.36% during the control period (2012–2022). The likelihood of a case occurring in rural areas was 1.30 times higher (95%CI: 1.18–1.44; p < 0.0001) than in urban areas. We observed a significant 72.13% drop in autochthonous cases in 2015 compared to the previous year. Only 76 autochthonous cases (6.48% of the total) occurred in rural zones in 2022. Overall, the trend reflects a strong decline, despite fluctuations throughout the years. Municipalities with the highest average API were located in the Amazon biome: Centro Novo do Maranhao (14.22), Boavista do Gurupi (14.06), Conceicao do Lago-Acu (10.96), Amapa do Maranhao (10.12), and Candido Mendes (9.58). From 2012 to 2022, 129 municipalities (59.45%) reported at least one malaria case. In the past three years, 138 municipalities (63.59%) recorded no autochthonous cases. Only nine municipalities reported more than 10 cases during this period (Figure 2C).

### Malaria in urban areas

Urban malaria accounted for 16.85% of all autochthonous cases. Comparing data from 2003 to 2022, urban cases decreased by 99.59%, with reductions of 82.64% from 2003 to 2011 and 96.70% from 2012 to 2022. The median number of urban cases decreased significantly, from 803 cases (95%CI: 338–1,705) in 2003–2011 to 17 cases (95%CI: 7–71) in 2012–2022, demonstrating a statistically significant reduction in both periods (p < 0.0001).

### Malaria in rural areas

The reduction in cases in rural areas from 2003 to 2022 was 99.07%, with decreases of 74.79% from 2033 to 2011 and 90.40% from 2012 to 2022. From 2003 to 2022, the median annual number of autochthonous malaria cases in rural areas was 622 cases (95%CI: 147–3,162); for 2003–2011 it was 4,017 cases (95%CI: 1,865–7,673), and for 2012–2022 it was 147 cases (95%CI: 77–526). These differences were statistically significant (p < 0.0001).

### Malaria in indigenous areas

All malaria cases in indigenous areas occurred within officially designated territories located in the Amazon biome. Case numbers fluctuated considerably, ranging from zero in 2013 and 2016 to 61.15% of rural cases in 2018, 50.65% in 2020, and 41.00% in 2019. Malaria in indigenous areas has increased since 2017; however, only three cases were reported in the last two years. From 2012 to 2022, the average number of cases in indigenous areas was 35 ± 51.150; with a median of five (95%CI: 0–98) and a maximum of 159 cases in 2018. In 2014, indigenous areas in the municipalities of Buriticupu and Santa Luzia accounted for most of the 39 registered cases. In 2017, villages within the Araguana, Jenipapo dos Vieiras, and Ze Doca municipalities recorded 98 cases, of which 77 occurred in the latter. In 2018, high case numbers were again recorded in Ze Doca (20 cases) and Jenipapo das Vieiras (124 cases). In 2019 and 2020, villages within Jenipapo das Vieiras municipality reported 38 and 29 additional cases, respectively. In 2021 and 2022, only one case was recorded in these areas.

### Imported cases

We consider only imported malaria cases in this section, focusing on probable sites of infection, time dynamics, and their contribution to the overall malaria burden in the state. The average annual number of imported cases was 1,344 (± 604), with a median of 1,338 (95%CI: 265-1,608). Before 2008, importations represented less than 30.00% of cases. However, starting in 2012, a significant shift occurred: imported cases surpassed autochthonous cases, reaching 94.00% of all cases by 2022. The odds of an imported malaria case in 2012–2022 compared to 2003–2011 were 7.97 times higher (95%CI: 7.63–8.33; p < 0.0001) (Table 1 and Figure 2). This result reveals decreased local transmission and increased introductions from external sources. Among imported cases, 39.59% originated from other Brazilian states and 60.41% came from other countries. Since 2020, domestic imports have outnumbered international ones, largely driven by mining and labor migration. In the past five years, 85.97% of all cases were imported, with domestic sources accounting for 67.39%, a sharp increase compared to 27.70% at the beginning of the series (OR: 5.39; 95%CI: 4.98–5.84; p < 0.0001). Most domestically imported cases originated from Para (52.31%) and Roraima (34.50%). International importations were mainly from French Guiana (55.93%), Guyana (24.72%), and Venezuela (15.16%), together representing nearly 95.00% of all international imported cases. These findings underscore the role of both internal and cross-border human mobility in sustaining malaria transmission, highlighting the need for coordinated, multisectoral actions across regions and national borders.

### Seasonality and epidemic peaks

**Figure 3:**
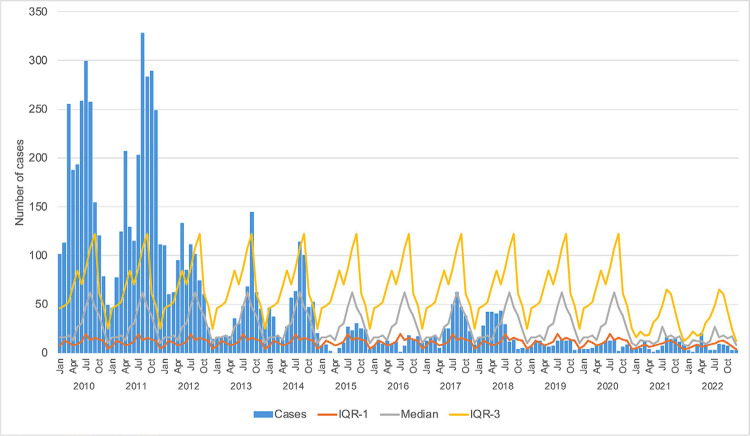
Endemic curve and autochthonous malaria cases per month in Maranhao State, 2010–2022. Monthly medians were calculated across all years (e.g., all January values from 2010 to 2022). The interquartile range (IQR) was computed using the 25th (Q1) and 75th (Q3) percentiles of monthly values. Epidemic peaks were defined as months exceeding Q3. Only autochthonous cases were considered for this analysis.

To avoid bias from the period when malaria was still highly endemic in the state, the endemic curve was built using data from 2010–2022. Based on the reported autochthonous cases, this study shows an increase in May and peaks between September and October, when the rainy season (January–March) ceases and the dry season (August–October) begins (Figure 3). From January to April, transmission remained low. Between 2010 and 2022, eight epidemic outbreaks were identified: six of short duration (mean of 52 days) and two longer ones, occurring between February and October 2010 and from March to December 2011. These larger outbreaks marked the end of the epidemic period. No outbreaks were detected after 2015, and subsequent cases remained within the endemic curve. Recently, the number of autochthonous cases has dropped so low that clear seasonal patterns have not emerged.

### Plasmodium spp. species

Regarding parasitic species, 9,388 (16.10%) infections were caused by *P. falciparum*, 48,807 (83.69%) by *P. vivax*, 126 (0.22%) were mixed infections (*P. vivax* + *P. falciparum*), 12 by *P. malariae*, and three additional mixed infections (*P. falciparum* + *P. malariae*) were recorded throughout the period. The annual mean number of *P. falciparum* cases was 469 ± 718 (95%CI: 133–806; median 26), whereas *P. vivax* averaged 2,440 ± 3,122 (95%CI: 979–3,902; median 765), (p < 0.0050). From 2003 to 2009, *P. falciparum* accounted for 17.74% of cases, but its proportion decreased to 6.01% in the post-epidemic period (2010–2022). In 2022, *P. vivax* predominated and accounted for 96% of reported cases. The odds of *P. falciparum* infection were 3.37 times higher during the epidemic period compared to the post-epidemic years (95%CI: 3.07–3.70, p < 0.0001) (Figure 2D).

### Age group and gender

**Figure 4:**
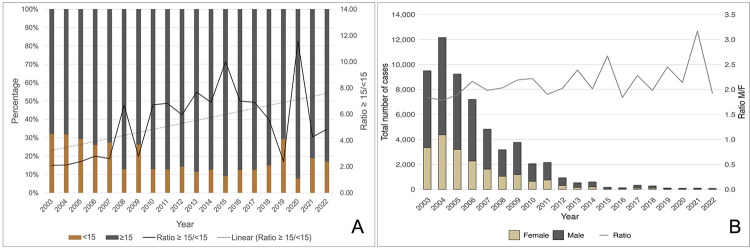
(A) Malaria cases by age groups and ratio of ≥ 15 to < 15 years; (B) Percentage and male-to-female ratio of malaria cases, 2003–2022.

During the study period, most malaria cases, 51,021 (75.57%), occurred in individuals over 15 years of age (including both autochthonous and imported). There was an increase from 59.10% in 2003 to 70.50% in 2022 in the 15–49 age group. Comparing 2003–2011 to 2012–2022, cases among individuals aged 15 or older increased from 72.38% to 86.82%. The relative risk of infection in this age group was 2.5-fold higher after 2011 (95%CI: 2.39–2.65; p < 0.0001), suggesting that transmission concentrated in adults, especially those of working age. Regarding sex, 66.07% of cases occurred among males, with a continuous male-to-female ratio of approximately 2:1. Male proportions were 64.80% in 2003and 65.80% in 2022, confirming consistent epidemiological patterns by sex (Figure 4).

### Annual parasitic incidence (API)

**Figure 5:**
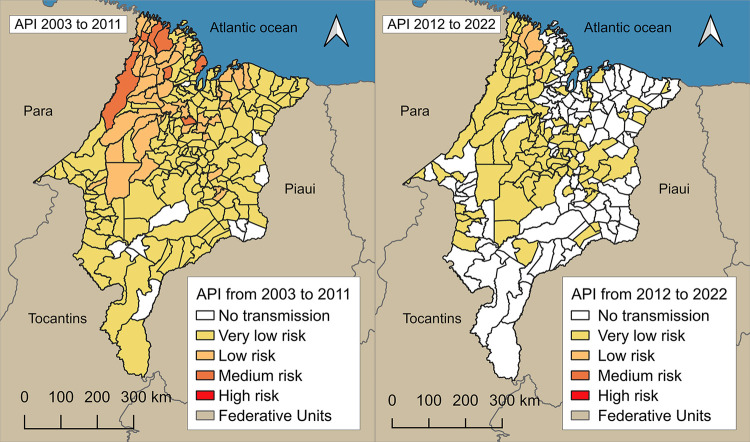
Cumulative Annual Parasite Incidence (API) from 2003 to 2022: (A) Average API per municipality, 2003–2011;

Historical data from Maranhao State revealed a significant drop in the API over the study period (p < 0.0001), indicating a marked reduction in malaria transmission. The average statewide API was 0.46 cases per 1,000 inhabitants, decreasing from 1.55 in 2003 to 0.01 in 2022. During the initial period (2003–2011), Centro Novo do Maranhao reported the highest burden with 4,457 cases (8.10%), with an average of 31 cases per 1,000 inhabitants, the highest recorded in the series. In this period, eight municipalities (3.69%) were classified as medium risk, 50 (23.04%) as low risk, 148 (68.20%) as very low risk, and 11 (5.07%) as no risk. In the subsequent period (2012–2022), Candido Mendes registered the highest number of cases (377). No municipality was classified as high or medium risk. Only five (2.30%) were classified as low risk, 98 (45.16%) as very low risk, and 114 (52.53%) reported no cases. The distribution by biome also reflected this trend (Figure 5). In the Amazon biome, 2,598 cases (80.10%) were reported, of which 6.02% were in low-risk municipalities, 55.42% in very low-risk municipalities, and 38.55% in municipalities without any risk. The Amazon/Cerrado transition reported 199 cases (6.10%), with 52% in very low-risk areas and 48.00% in no-risk areas. Cerrado municipalities documented 448 cases (13.80%), of which 34.83% were in very low-risk areas and 65.14% in no-risk areas. Only 10 cases were reported in the Cerrado/Caatinga transition zone in the early part of the series, and none have been recently reported.

(B) API from 2012 to 2022.

In the past three years (2020–2022), the municipalities of Jenipapo dos Vieiras, Candido Mendes, and Amapa do Maranhao were the only ones that consistently remained in the low-risk category. During this period, 98 municipalities (45.16%) were categorized as very low risk, while the remaining 114 (52.53%) reported no risk. Overall, only 47 municipalities (21.66%) recorded malaria cases, whereas 170 (78.34%) did not. Among those without cases, 52.69% were located in the Cerrado, 34.13% in the Amazon, 13.17% in the Amazon/Cerrado transition, and only 0.59% in the Cerrado/Caatinga transition. The analysis also showed that the likelihood of malaria occurrence was 4.5 times higher in municipalities located in the Amazon or in the Amazon/Cerrado transition zone compared to those situated exclusively in the Cerrado (95% CI: 1.61–13.2; p = 0.0032).

## DISCUSSION

In recent years, the world has renewed its commitment to eliminating malaria^1^. In 2015, Brazil was among the countries that achieved the reduction target proposed by the WHO, with a 76.70% decrease in malaria cases compared to 2000. The lowest number of cases in the last 35 years was recorded in 2016, with 128,746 cases^9,10^. This progress led the Brazilian Ministry of Health to launch the *P. falciparum* Malaria Elimination Plan in 2015, which was later ratified in 2022 as the Brazil Malaria Elimination Plan. This national plan aims to eliminate *P. falciparum* malaria by 2030 and all species by 2035^9,10,18^. Although Maranhao State has historically presented lower malaria incidence rates compared to other Amazonian states, the disease has remained locally relevant in rural and vulnerable areas, especially in earlier periods, due to environmental and socioeconomic factors^3-5^. Our study showed a remarkable 99.16% reduction in autochthonous cases, from 9,102 in 2003 to only 76 in 2022, surpassing the target agreed upon with the Ministry of Health (176 cases in 2022)^9,10^. During the same period, Brazil registered a 67.27% decrease in cases^2,6,10^. Currently, Maranhao is considered a very low epidemiological risk area, with an API of 0.01 cases per 1,000 inhabitants, and is progressing steadily towards elimination. Although autochthonous cases have decreased, we observed a significant rise in imported malaria. In the past five years, 84.00% of reported cases were imported, indicating that malaria transmission in the state is now mainly influenced by the importation of cases. Compared to the beginning of the study period, the likelihood of an imported case occurring in the last decade was seven times higher. Furthermore, the characteristics of imported cases have changed over time. Initially, 50.00% to 86.00% of cases were acquired abroad; however, this proportion dropped to 32% and 15% by 2022^9^. Many of these international cases originated from countries within the Guiana Shield. The literature indicates that Maranhao is among the Brazilian states with the highest migratory flows to illegal mining regions, such as French Guiana and Suriname, where malaria represents a major public health issue due to high transmission rates and limited access to healthcare services^3,18,19^. Douine *et al*.^19^ outlined the sociodemographic profile of miners working illegally in French Guiana, showing that 93.80% were Brazilian, of which more than half (55.70%) were from Maranhao State. Silva *et al*.^20^ reported that one-third of miners engaged in illegal activities in French Guiana frequently returned to Brazil, particularly during year-end festivities. However, not all imported malaria cases can be attributed to French Guiana. A substantial proportion of cases originating from Guyana, Venezuela, and Suriname reflects intense movement of miners within the Guiana Shield, a well-recognized malaria hotspot in the Americas. This setting underscores the pressing necessity for global collaboration to formulate coordinated strategies to eliminate malaria across the Guiana Shield^21^. Infections originating from neighboring Brazilian states increased, while, in contrast, imported cases from other countries decreased. This shift may be related to a considerable rise in illegal mining activities in the Brazilian Amazon, particularly within indigenous lands and forest reserves. Data from the Ministry of Health in 2022 show that 900 malaria cases in Maranhao State were associated with mining, accounting for 79.37% of the total cases that year^9^. These data indicate the emergence of a novel pattern of imported malaria influenced by internal migration. In this dynamic, individuals travel to mining areas in endemic Brazilian regions and subsequently return to their municipalities, thereby disseminating the parasite to surrounding areas^22^. This context underscores the need for municipalities to strengthen epidemiological surveillance to prevent the reintroduction of malaria. All newly reported cases must be thoroughly investigated to confirm the diagnosis and identify the probable site of infection. Once confirmed, control measures must be promptly implemented to prevent further infection. Over the past two decades, all municipalities in Maranhao State have had a decline in autochthonous malaria, with most now categorized as low or very low epidemiological risk, predominantly restricted to the Amazon biome. Urban malaria showed a notable reduction, constituting 16.85% of total cases and ranging from 22.77% to 3.96%. In rural regions, only nine municipalities documented more than 10 cases in the past five years, underscoring the necessity of enhancing surveillance to achieve complete elimination throughout the state. Although this study follows the national urban-rural classification, it is important to note that malaria in Amazonian urban settings often occurs in localities with poor living conditions, intense population mobility, and severe environmental degradation^23^. A study conducted in the Mancio Lima and Rio Branco municipalities, in Acre State, revealed both imported and autochthonous malaria cases, with *Plasmodium* spp. strains showing genetic similarities across different neighborhoods^24^. In Vila Candelaria, a peri-urban area, persistent mosquitoes breeding sites and high proportions of undiagnosed infections hinder accurate diagnostics, while the mobility of laborers between urban and rural territories is a key factor for increased transmission^25^. The ecological, social, and infrastructural configurations that further complicate malaria dynamics in peri-urban and urban sectors of the Amazon highlight the need for traditional yet context-specific approaches for both control and surveillance. Thus, this framework underscores the challenges of managing malaria in peri-urban and urban areas of the Amazon, where environmental, social, and infrastructural vulnerabilities are often intensified, requiring different preventive and control methods. Over the past three years, 167 municipalities (77.06%) reported no autochthonous malaria cases, suggesting they might have reached elimination; however, continuous surveillance remains essential to detect new cases^17^. In low-incidence settings, outbreaks are often associated with increases in vector populations and reductions in control measures^1^. However, regions approaching elimination must maintain surveillance, as imported cases can trigger reintroductions if not promptly identified and contained^9^. Surveillance programs should focus on key areas and transmission dynamics to guide appropriate actions, including studies in municipalities that have not reported recent cases^26,27^. This method helps identify areas where interventions are necessary and whether surveillance strategies require adjustment^1^. Should new cases arise or persist, enhanced investigations and increased surveillance may be required^7^. Increased surveillance, prudent use of insecticide-treated nets (ITNs), and better diagnostic capacity, particularly for *P. vivax*, which persists despite the decrease in *P. falciparum*, have all contributed to the notable decline of malaria in Maranhao State^28,29^. Environmental alterations, including deforestation and the expansion of forest margins, have favored *Nyssorhynchus darlingi* in specific transitional ecosystems^30^. This vector does not flourish uniformly across all degraded areas, as its prevalence depends on particular ecological characteristics such as the availability of larval habitats and climatic circumstances^31^. Socioeconomic vulnerabilities and patterns of labor mobility further impact transmission, underscoring the need for integrated methods that combine ecological, social, and territorial strategies. Despite malaria’s robust correlation with poverty and Maranhao’s status as one of the most socioeconomically disadvantaged states in the Brazilian Amazon, the incidence for this disease has markedly decreased. This apparent contradiction can be explained by ongoing monitoring, improved testing methods, careful use of insecticides, and training for local health workers, particularly in areas with limited infrastructure^5,32^. Additionally, the Bolsa Familia program, a federal initiative providing income redistribution and social benefits to low-income families, has served as a platform for intersectoral health interventions and has indirectly promoted food security^33^. These findings illustrate that the most effective approach to malaria control combines integrated and localized public health policies, even in contexts of longstanding poverty. In circumstances where the ongoing surveillance is inadequate or fails to identify specific risks, spot checks must be conducted^1^. Our study showed that adults, particularly men aged 15 to 49, were the most affected, with individuals over 15 years of age exhibiting a 2.5-fold higher risk of illness compared to those under 15^34,35^. The sex ratio remained consistent throughout the study period at approximately 2:1. The data indicate a significant correlation between malaria and occupational exposure in Maranhao State, particularly in mining, extractive industries, and other predominantly male occupations, as noted in other Amazonian regions^36^. In some indigenous areas, minor variations in case numbers were observed in relation to localized outbreaks. Such circumstances underscore the importance of detailed investigations and timely, targeted interventions to prevent the recurrence of transmission^9,10,21^. Regarding parasitic species, *Plasmodium vivax* was the main species in the state, with 83.69% of all cases over the study period and 96.00% in 2022. Although *P. falciparum* represented only 6.60% of cases in 2022, ongoing monitoring remains essential, as the influx of miners from Guiana Shield poses a persistent risk to the introduction of drug-resistant pathogens^37^. The WHO advises categorizing territories into various risk zones with analytical classifications such as receptivity and case importation^38,39^. These categories enable health authorities to distinguish ecological factors influencing vector distribution (receptivity) from sociodemographic ones connected with human mobility. This stratification supports the development of a microstratification model for malaria foci, which is essential for eliminating malaria in low-transmission regions^22,38,39^. This study is limited by its reliance on secondary data from the Brazilian surveillance system, particularly for identifying socio-environmental determinants. Underreporting is likely, especially in regions of illicit mining. Nevertheless, the issue appears to be of lower concern in other regions due to PNCM guidelines, which mandate prompt diagnosis, free treatment, and compulsory case reporting. In Maranhao State, these protocols seem to have been consistently applied, contributing to the increased data reliability.

## CONCLUSION

The study showed a considerable decrease in malaria cases in Maranhao State over the last 20 years, leading to its classification as a low or very low epidemiological risk area. No new outbreaks have occurred since 2015, and due to the low number of autochthonous cases, clear seasonal patterns are no longer evident. Analysis of the entire period also revealed an increasing proportion of cases among adult men, who currently account for most infections, unlike patterns found elsewhere in the Brazilian Amazon. Although imported cases have always contributed to the disease burden, most reported cases since 2012 come from outside the state, and in the last four years of the data series, over 80% of cases were imported. A shift in the sources of imported cases was also observed: early in the study, most cases came from countries in the Guiana Shield, whereas currently, the majority originate from other Brazilian municipalities. Transmission appears to be predominantly influenced by population mobility, including illicit mining, which drives case imports. These changes are concerning, as they could reverse previous gains in malaria control and trigger a resurgence in a state that was close to elimination.

## Data Availability

The complete anonymized dataset supporting the findings of this study is included within the article itself.
